# Supplementary material for: Qualitative Insights into Vaccine Uptake of Nursing Staff in Long-Term Care Facilities in Finland
Source: Vaccines (Basel). 2023 Feb 23;11(3):530. doi: 10.3390/vaccines11030530 (PMC10056830; doi:10.3390/vaccines11030530)
Supplement: Supplementary file 1 [file vaccines-11-00530-s001.zip › S1_Question Guide Nursing Staff.pdf]

Question guide for interviews with the nursing staff

	We would like to start the interview by asking about factors that influenced your decision not to take the vaccine.
Knowledge	What about your knowledge? Did it influence your decision?<br>Probes: Do you feel that you have received enough information to decide on the vaccine? Did you have some information that made you decide not to take the vaccine?
	What is the source of information that matters to you most when deciding to take the vaccine?<br>Probes: Family and friends, official information, traditional media, social media & social media influencers other influencers
Skills	Dis you perceive yourself as skilled enough to make the decision for yourself not to take the vaccine?
Beliefs about capabilities	Did you perceive yourself as capable of taking the vaccine?<br>Probes: physical health, reproductive health, mental health
social and professional role and identity	How does your profession influence on your decision not to take the vaccine?
Social influences	With whom did you discuss COVID-19 vaccination?<br>Probes; family, friends, colleagues, managers<br>How did these discussions influence your decision not to take the vaccine?
Beliefs in consequences	What kind of consequences did you perceive with the COVID-19 vaccine?<br>How did these perceptions influence your decision not to take the vaccine?
Optimism	Do you believe that the vaccine can halt the pandemic? If yes, explain how?
Reinforcement	What kind of management support or encouragement did you get for decision-making to take the vaccine?
Intention	Did you consider it at any point during the pandemic taking the vaccine? If yes, tell me about you're the time you considered it and the turning point when you decided not to take it.
Goal	Was taking the vaccine a goal?<br>Probe: At any point of time
Behavioral regulations	Did you make any concrete plans that helped you to make the decision?<br>Probes: Making a list of pros and cons, consulting experts
Emotions	Did you experience strong emotions that made you decide not to take the vaccine? Tell me about them.
Psychological influences	Was your mental well-being influenced by the COVID-19 vaccine? if yes explain more.
Environmental context & resources	What kind of practical matters influenced your decision not to take the vaccine?<br>Probes: vaccine booking system, vaccine place, vaccine time, type of vaccine offered, staff attitude
