# Supplementary material for: Qualitative Insights into Vaccine Uptake of Nursing Staff in Long-Term Care Facilities in Finland
Source: Vaccines (Basel). 2023 Feb 23;11(3):530. doi: 10.3390/vaccines11030530 (PMC10056830; doi:10.3390/vaccines11030530)
Supplement: Supplementary file 1 [file vaccines-11-00530-s001.zip › S2_Question Guide Management.pdf]

Question guide for interviews with the management

	We would like to talk to you about your staff members who refused to take the COVID-19 vaccine and their reasons for that.
Knowledge	What do you know about their knowledge? Do you think it had an impact on the decision not to take the vaccine? about your knowledge? Did it influence your decision?<br>Probes: Did they have enough information to decide on the vaccine? Did some of the information they received make them not take the vaccine?
	What is the source of information that matters to your staff mostly when deciding to take the vaccine?<br>Probes: Family and friends, official information, traditional media, social media & social media influencers other influencers
Skills	Do you think your staff perceives themselves as skilled enough to make the decision for themselves not to take the vaccine?
Beliefs about capabilities	Do you think your staff perceives themselves as capable of taking the vaccine?<br>Probes: physical health, reproductive health, mental health
Professional role	How do you think the professional role of the nursing staff influences their vaccination decision? What about those who have refused the vaccine?
Social influences	With whom does your nursing staff discuss COVID-19 vaccination?<br>Probes; family, friends, colleagues, managers<br>How do you think these discussions have influenced their decision not to take the vaccine?
Beliefs in consequences	What kind of consequences do you think your staff perceives with the COVID-19 vaccine?<br>How did these perceptions influence their decision not to take the vaccine?
Optimism	Does your staff believe that the vaccine can halt the pandemic? If yes, explain how?
Reinforcement	What kind of management support or encouragement did your staff get in the decision-making to take the vaccine?
Intention	Do you think there were times during which your staff members who have not taken the vaccine, considered doing so? If yes, what do you know about those individuals and their thinking?
Behavioral regulations	Do you know if your staff made any concrete plans that helped them to decide not to take the vaccine?<br>Probes: Making a list of pros and cons, consulting experts
Goal	Do you think
Emotions	Did you observe strong emotions among the staff members who refused the vaccine when discussing the topic? Tell me more about it.
Psychological influences	Was mental well-being influenced by the COVID-19 vaccine? if yes explain more. Do you think this influence the decision of some staff members not to take the vaccine?
Environmental context & resources	Were there any practical issues that influenced the staff decision not to take the vaccine? Probes: vaccine booking system, vaccine place, vaccine time, type of vaccine offered, staff attitude
